# Laparoscopic management of large colonic lipoma with atypical presentation: A case report

**DOI:** 10.1016/j.ijscr.2024.110281

**Published:** 2024-09-12

**Authors:** Mohamad EL Haress, Mohamad Siblini, Mohamad Zaatari, Salim Albast, Oubaida Elkhatib

**Affiliations:** Department of Surgery, Makassed General Hospital, Beirut, Lebanon

**Keywords:** Urine urgency, Polyp, Lipoma, Colonic lipoma, Colonoscopy, Computed tomography, Case report

## Abstract

Colonic lipomas (CL) are a rare condition that typically causes symptoms in only a minority of patients. When large lipomas occur, they often necessitate extensive surgery, which carries significant risks.

**Case presentation:**

We present a case of a female patient who experienced abdominal pain and urinary urgency due to a large, 80 % obstructive lipoma in the descending colon. On abdominal Computed tomography scan, a 3.8 cm lesion with fatty density and no solid components was identified. Given its benign nature, our approach aimed to preserve the colon. This involved performing laparoscopic excision of the lipoma after marking the base of the mass with a methylene blue dye few hours prior to surgery. The patient recovered well postoperatively, with an excellent outcome, and was discharged home on the third day after surgery.

**Discussion:**

Colonic lipomas are a rare finding in the gastro-intestinal tract, they are benign in nature and can cause a variety of symptoms. The diagnostic pathway can be challenging due to the broad presentation and the variable onset of symptoms. Using multiple imaging modalities (invasive and non-invasive methods) can help narrow down the diagnosis and facilitate the treatment course.

**Conclusion:**

Our review of literature indicated that Descending Colon lipoma is rare. With nonspecific symptoms imaging modalities such as computed tomography was used in conjunction with Colonoscopy to further delineate origin and pathology. The treatment depends on the patient's condition as well as the size and position of the tumor.

## Highlights

• Colonic lipomas are a rare condition that occurs in a minority of patients.
• Clinical findings have a wide variety of presentations and onsets.
• Using multiple invasive and non-invasive imaging modalities can help in the diagnosis and management.
• The treatment plan depends on the size of the lipoma and the condition of the patient

## 1 Background

Colonic lipomas are one of the asymptomatic diseases if they were small and can have a broad range of symptoms if they were large enough to cause them. The most common symptoms found in large CLs are abdominal pain (being acute, chronic, intermittent, or colicky in nature), Rectal bleeding, constipation periods followed by diarrhea or obstipation.

This case of colonic lipoma had a very unusual presentation that was treated and had excellent post operative stay and no short-term complications.

The usual treatment of CLs can range from endoscopic excision to robotic assisted excision. The laparoscopic approach was used in this case after tattooing its base with methylene blue dye with the intent of preserving the colon.

## 2 Case report

Presentation: This is the case of a previously healthy 34-year-old female patient, a smoker allergic to penicillin, presenting with intermittent right lower abdominal pain, colicky in nature. Along with urine urgency that started 3 weeks before presentation. The pain is only temporarily relieved by pain medications. The patient reported experiencing constipation (1 bowel movement per week) for the past 4 weeks. In addition, the patient denied any change in oral intake, weight loss, nausea, vomiting, melena, hematochezia, hemoptysis, headache, fever, chills, urinary symptoms, or any other symptoms.

**Fig. 1:**
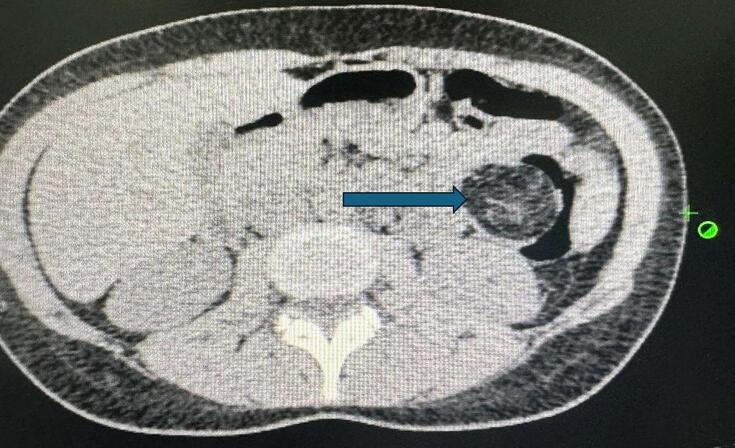
CT scan of the abdomen showing: a round well-defined 3.8 × 3.4 cm mass in the proximal aspect of the descending colon, with most likely lipid in nature.

**Fig. 2:**
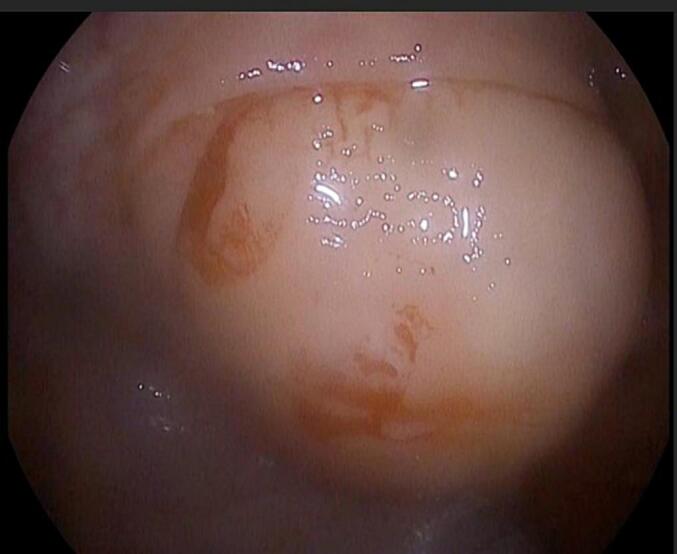
Colonoscopy showing a yellow isolated solitary mass with normal overlying mucosa. (For interpretation of the references to colour in this figure legend, the reader is referred to the web version of this article.)

Investigation: The history reveals that the patient sought medical advice from a gastroenterologist and underwent a CT scan, which revealed a 3.8 × 3.4 cm lesion, located approximately 20 cm distal to the splenic flexure in the descending colon, arising from its wall, with predominant fat density that occupied approximately 80 % of the lumen (Fig. 1). Subsequently, the patient underwent upper endoscopy and colonoscopy, which revealed a 4 cm solitary bulge with normal overlying mucosa located 32 cm from the anal verge at the splenic flexure (Fig. 2). The bulge had a smooth, regular appearance and was biopsied, and blue dye was injected underneath the base of it. The biopsy results showed a hyperplastic polyp with normal surface villi and no dysplasia.

**Fig. 3:**
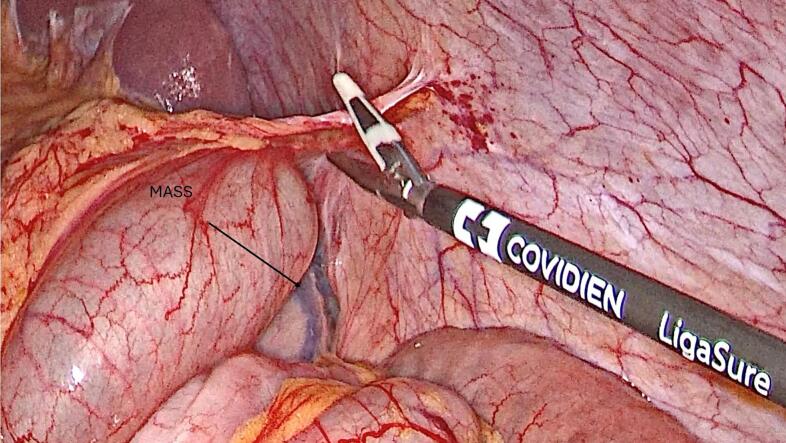
Dissection around the descending colon, revealing the tattooed area below the mass.

**Fig. 4:**
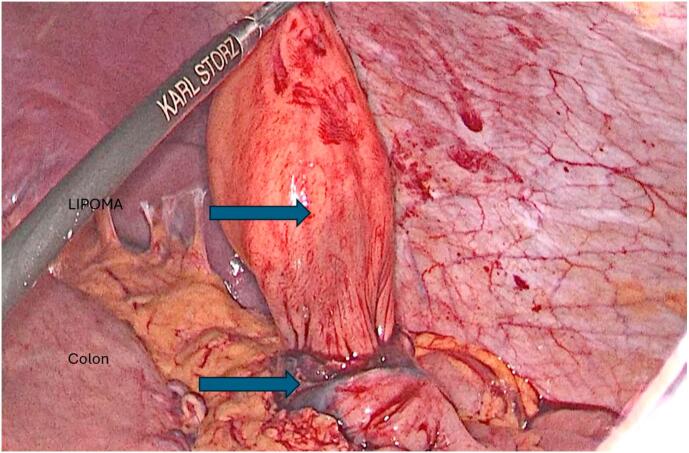
Delivering of the mass through the opening of the colon and excision at its base.

**Fig. 5:**
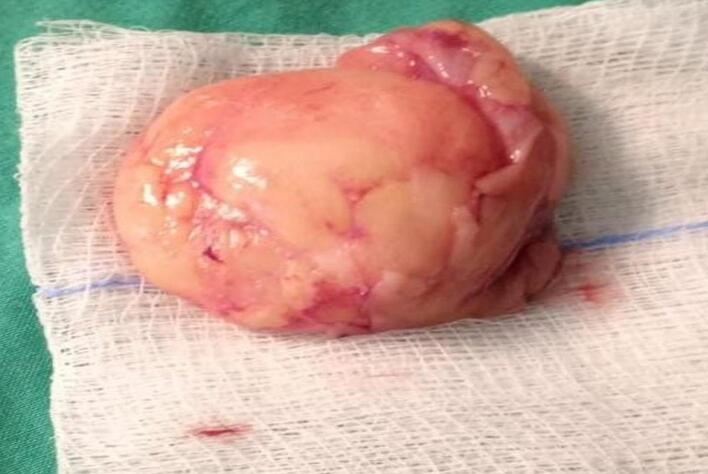
Macroscopic view of the excised lipoma.

Management: Endoscopic mucosal resection was deemed non-feasible due to the size of the mass. Laparoscopic excision of the lipoma after delivering it outside the colon was done using the following technique: Under general anesthesia, the patient was placed in a traditional Lloyd-Davies Position, with the left arm abducted and the right arm positioned alongside the body. The operating table was tilted towards the right and ranged between Trendelenburg and reverse Trendelenburg positions depending on the various operative steps. A 3-mm incision was made under the umbilicus and a 12-mm trocar was inserted using the “hasson Technique”. The abdomen was insufflated using carbon dioxide to a target pressure of 12-14 mmHg. under direct supervision with laparoscopic assistance, a second 12-mm trocar and two 11-mm trocars were inserted into the right-middle subcostal area and frontal axillary lines in the bilateral regions of the abdomen respectively. The intra-abdominal cavity was explored for any irregularities and to identify the tattooed part of the colon. A vertical incision was done to the anterior surface of the colon at the level of the tattooed area. The colonic lumen was entered using the camera scope. The lipoma was delivered outside the colon from the inside of the posterior wall. And using Endo-GIA 60, the lipoma was excised along with the mucosa with no colonic layers disruption (Fig. 3, Fig. 4). No edges were sutured. Using V-lock 3.0, the anterior colonic wall incision was closed, and a negative leak test was established. A graham patch was fixed on the sutured colon. One Hemovac was inserted into the abdomen. The fascia and skin were closed using 2.0 Vicryl and 4.0 Monocryl respectively. The patient had a soft post operative hospital stay and was sent home 3 days after surgery. The macroscopic image of the mass is shown in (Fig. 5). The last test of histopathological examination revealed submucosal mass with regular overlying mucosa *showing normal surface villous shape with no dysplasia*. A search on “PubMed” for words like colonic lipomas gave ninety-nine articles. Of these, twenty-eight recent case reports spanned the last decade. Tumor measures ranged from 2 to 5 cm, and the main treatment was endoscopic resection.

## 3 Discussion

Lipomas are a rare finding in the GI tract. They are benign in nature, with an incidence ranging from 0.035 % to 4.4 % compared to other types of polyps in the colon [1].

Clinical findings defer from patient to patient due to the size of the lipoma, the place from which it arises is rarely relevant to the symptoms found.

Symptoms are equivocal and have an extraordinarily long and unusual onset of appearance, since they are related to the size of the tumor. Some patients present with symptoms of obstruction, others with bleeding and subsequent anemia [4]. Intussusception can arise in patients with exceptionally large lipomas. That can render the diagnosis difficult to achieve preoperatively without the proper experience and tools. By reviewing the literature, we did not find any case of colonic lipoma presenting with urinary urgency.

Using available imaging modalities (CT scan, barium enema and colonoscopy) can help identify the cause of the symptoms and guide the physician to a definitive diagnosis [3].

.Barium enema can help finding the preliminary affected region of the colon by illuminating the part of the intestine distal to the tumor, and staying clear of the region proximal to it if the tumor is large enough to cause complete obstruction of the intestinal lumen. If the tumor cannot obstruct the full radius of the bowel, the barium could leak past the lipoma into the proximal intestine depending on the size of the available lumen left unobstructed. This imaging modality can be ineffective if the lipoma is too small to affect the flow of the barium in the bowels.

Using CT scan, the lipoma can be seen as a uniform, dense tumor with smooth borders. But it can be difficult to differentiate it between the other malformations if its small [3].

The best direct view of the tumor can be achieved by colonoscopy, since the tumor can be visualized as “tenting of mucosa.” When pressed by the endoscope, the lipoma can be easily indented, and it can spring back to its normal shape when the force is withdrawn. Excision is not recommended in these patients, since the lipoma was large, below the mucosal wall and the risk of post-biopsy bleeding, or perforation is increased [2].

In our case, we resorted to the laparoscopic technique with no intent of excising any part of the colon. The size of the lipoma was defined by computed tomography. Colonoscopic resection was discouraged in this case due to the increased risk of bleeding based on the size of the lipoma. Opne surgery was considered, with its advantage on having a wider field to work with less intraoperative complications compared with laparoscopic techniques. However, the increase in hospital stay (intraoperative and postoperative) has an increased risk of complications, like late ambulation, nosocomial infection. Furthermore, the daily hospital charges are greater due to the increased stay in hospital. Laparoscopic resection was the only viable option given the advantages over other techniques. The identification of the tumor from the outside of the colon was straightforward due to the usage of methylene blue tattooing at the base of the lipoma endoscopically.

The pros of this laparoscopic technique were the ability to preserve the colon integrity without anastomosis, the decreased hospital stay post-operatively, and the benefit of decreased complications found in laparoscopic surgeries in general. On the other hand, this laparoscopic technique had its own setbacks due to the scarcity of literature review about these types of cases.

## 4 Conclusions

Our review of literature indicated that Descending Colon lipoma is rare. With nonspecific symptoms imaging modalities such as computed tomography was used in conjunction with Colonoscopy to further delineate origin and pathology. The treatment depends on the patient's condition as well as the size and position of the tumor.

## List of abbreviations

CL: colonic lipoma
CT: computed tomography

## Ethical considerations

Written informed consent was obtained from the patient for publication of this case report and accompanying images. A copy of the written consent is available for review by the Editor-in-Chief of this journal on request.

The work reported has been in line with the SCARE criteria [5].

## Ethical approval

This study is exempt from ethical approval in our institution, since the case was already discharged from the hospital and case reports are considered to not need ethical approval from Makassed General Hospital IRB.

## Funding

No source of funding was needed.

## Author contribution

Mohamad El Haress M.D.: writing (original draft).

Mohamad Siblini M.D.: Conceptualization, supervision, project administration.

Mohamad Zaatari M.D.: Validation, project administration.

Salim Albast M.D.: Writing (original draft).

Oubaida Elkhatib M.D.: writing (review and editing).

## Guarantor

No guarantors were needed.

## Registration of research studies

No registries were behind this research project.

## Conflict of interest statement

No conflict of interest was found in this case.
